# Remote management of anaemia in patients with end-stage kidney disease using a wearable, non-invasive sensor

**DOI:** 10.1093/ckj/sfae375

**Published:** 2024-11-22

**Authors:** Amy Steig, Forrest Miller, Samir Shreim, Jake Wilcox, Carole Sykes, David Whittaker, Rajesh Sivaprakasam, Samit Gupta, David Kuraguntla

**Affiliations:** Alio, Inc., Broomfield, CO, USA; Alio, Inc., Broomfield, CO, USA; Alio, Inc., Broomfield, CO, USA; Alio, Inc., Broomfield, CO, USA; Clinical Strategy Group, San Francisco, CA, USA; The Greenbrier Clinic, White Sulphur Springs, WV, USA; Barts Health NHS Trust, London E1 1BB, UK; Alio, Inc., Broomfield, CO, USA; Alio, Inc., Broomfield, CO, USA

**Keywords:** anaemia, biomarkers, chronic renal failure, clinical trial, ESRD, haemoglobin, remote patient monitoring, vascular access

## Abstract

Anaemia is a prevalent complication in patients with end-stage kidney disease (ESKD) undergoing haemodialysis. This study evaluates the accuracy of the Alio SmartPatch™, a non-invasive remote monitoring device, in measuring haemoglobin (Hb) and haematocrit (Hct) levels in haemodialysis patients by comparing its results with standard blood-based laboratory methods. The results from 116 patients across multiple sites in the USA and the Kingdom of Jordan show that SmartPatch measurements align closely with standard blood-based laboratory methods, meeting clinically acceptable limits of agreement. The current standard methods of Hb and Hct measurements are invasive and require visits to clinical sites, whereas the FDA-cleared SmartPatch offers non-invasive measurement of these parameters up to 240 times per month, thereby enhancing personalized care and patient engagement. This study demonstrates the potential of remote monitoring technologies, such as the SmartPatch, to improve the management of ESKD-associated anaemia. Further research is warranted to evaluate the device's long-term outcomes and its impact across diverse patient populations.

KEY LEARNING POINTS **What was known**:

- End-stage kidney disease (ESKD)-induced anaemia is a significant complication in patients with ESKD and warrants regular monitoring for haemoglobin and haematocrit, which can improve timely optimization of treatment.
- Existing monitoring methods, including point-of-care devices, are invasive, resource-intensive, or unsuitable for longitudinal, remote use.

**This study adds**:

- The SmartPatch provides accurate, non-invasive haemoglobin and haematocrit measurements comparable to laboratory methods.
- The FDA-cleared SmartPatch is the only clinical grade device with the ability to measure multiple metrics, including haemoglobin, haematocrit, abnormal potassium, heart rate, auscultation, and skin temperature, in patients with ESKD.

**Potential impact**:

- Adoption of the SmartPatch in care delivery offers opportunity to develop remote renal care of ESKD-induced anaemia.
- The ability to closely track haemoglobin and haematocrit fluctuations potentially allows healthcare providers to optimize iron and erythropoietin-stimulating treatment, thereby potentially reducing anaemia-related complications and improving long-term health.

## INTRODUCTION

The incidence of CKD is increasing worldwide. Based on a recent paper published in *Nature*, CKD affects over 850 million individuals worldwide, exceeding 10% of the world's population [1]. Anaemia is a known complication of CKD that affects the majority of patients with end-stage kidney disease (ESKD). Impaired production of erythropoietin by the kidneys is considered the major cause of anaemia, and worsens as the disease progresses [2, 3]. Anaemia results in a deficit in oxygen delivery to all parts of the body. Initially, the body compensates for the reduced oxygen supply by increasing the heart rate [3]. If unrecognized or untreated in a timely manner, increased heart rate could lead to ventricular hypertrophy, resulting in cardiac function deterioration and thus further renal hypoperfusion, which in turn activates both the sympathetic nervous system and the renin–angiotensin–aldosterone system. Together, these sequelae further impair renal function, becoming a vicious cycle known as cardiorenal anaemia syndrome (Type IV) [4]. Anaemia in patients with CKD significantly increases the risk of morbidity and mortality. It also heightens their procedure-related risk for surgical interventions such as dialysis access procedures and renal transplantation. Thus, timely diagnosis of anaemia and continued monitoring are essential to developing a dedicated patient management plan.

The most recent Kidney Disease: Improving Global Outcomes (KDIGO) guidelines recommend a regular assessment of haemoglobin (Hb) levels in patients with CKD, increasing the frequency as kidney function declines [5]. Based on the guidelines, anaemia treatment starts with a trial of iron therapy. The target Hb level is 10–12 g/dL in CKD patients [5]. Anaemia control is clinically important, and if iron supplementation is not successful, then erythropoiesis-stimulating agent (ESA) therapy is warranted. The KDIGO guidelines support the initiation of ESA therapy in patients with Hb concentrations <10.0 g/dL. However, the dosage range and high cost of ESA drugs must be optimized based on an accurate picture of Hb levels and the target range. Unnecessarily low or high ESA dosing levels are associated with poor outcomes, as shown in the Correction of Hemoglobin and Outcomes in Renal Insufficiency trial [6]. The incidence of the composite primary outcome (death, congestive heart failure, stroke, and myocardial infarction) was significantly higher in patients with the Hb target value of 13.5 g/dL than in those with a target value of 11.3 g/dL [6].

Current Hb level monitoring practice often follows the KDIGO, Renal Association, European Renal Association, or National Institute for Health and Care Excellence, which recommend Hb monitoring every 2–4 weeks for patients starting ESA therapy and every 1–3 months for those who are stable on an ESA or intravenous iron therapy [5, 7, 8]. These guidelines are designed to balance the costs of testing with the benefits of timely interventions to improve patient outcomes. However, the recommended testing frequency may not adequately address the Hb variability in patients with CKD-related anaemia, which is often described as ‘haemoglobin cycling’ [9–11]. Haemoglobin cycling refers to the cyclical fluctuation in Hb levels, which is influenced by individual factors such as variable responsiveness to ESAs, inflammatory processes, and nutritional deficiencies [9, 12]. These fluctuations complicate the management of anaemia as the Hb levels are difficult to maintain within a targeted range, leading to suboptimal treatment responses and increased cardiovascular risks, which significantly affect patient well-being [13]. Ebben *et al*. highlighted this issue and reported, in a large cohort of >150 000 patients with ESKD monitored for 6 months, that only 6.5% maintained a target Hb range of 11–12.5 g/dL, with nearly 90% experiencing Hb fluctuations beyond the target boundaries [14]. The study found that patients with persistently low Hb levels had the highest degree of comorbidity and hospitalization, consistent with previous studies. Interestingly, those categorized in the high-amplitude fluctuation group, which experienced low, target-range, and high Hb levels within the 6-month period, had degrees of comorbidity and hospitalization similar to those near the lower boundary of the target range [14]. Given the increasing incidence of CKD and the growing demands on healthcare providers to deliver high-quality, timely care, there is a pressing need for more adaptable and less invasive approaches to effectively manage anaemia in CKD patients.

Traditional monitoring methods for anaemia in patients with CKD typically warrant periodic blood draws. This approach not only provides infrequent snapshots of a patient's health status but also involves delays in receiving laboratory results, thereby adding complexity to timely anaemia management. The resource-intensive nature of hospital visits for anaemia monitoring further increases the overall costs and burdens on healthcare systems [15]. To address these challenges, various point-of-care devices have been developed, yet each presents its own limitations and remains largely impractical for home use. For example, the Crit-Line™ (Fresenius Medical Care NA, Waltham, MA, USA) monitor, which measures Hb levels, must be directly connected to haemodialysis machines, confining its use to clinical settings during specific dialysis sessions [16]. HemoCue® (HemoCue, Angelholm, Sweden) devices offer more frequent testing flexibility but still require invasive finger pricks, which can be cumbersome and deter patient compliance [17]. Their design also hinders remote use due to the invasive nature and necessity for a fresh blood sample. Whilst Masimo's (Pulse CO-Oximetry™; Masimo Corp., Irvine, CA, USA) non-invasive devices do not require direct blood sampling, patients need to remain tethered to a monitor, limiting their utility for remote or long-term monitoring—critical features for effectively managing chronic conditions. These limitations highlight the urgent need for innovative solutions that enable longitudinal, non-invasive, and patient-friendly Hb level monitoring to enhance anaemia management in patients with CKD [18].

The Alio SmartPatch™ represents a breakthrough in anaemia management in CKD. As an FDA-cleared device, the SmartPatch offers improvement over existing technologies by enabling real-time, non-invasive, and remote monitoring of multiple health parameters, including the world's first non-invasive monitoring of abnormal potassium via a functioning arteriovenous (AV) access [19]. Alongside abnormal potassium, the SmartPatch tracks Hb, haematocrit (Hct), heart rate, auscultation, and skin temperature. By delivering longitudinal data, the SmartPatch has the potential to facilitate timely interventions and supports a personalized approach to anaemia management, thereby enhancing patient outcomes and quality of life.

In this study, we evaluated the accuracy of the SmartPatch in measuring Hb and Hct levels in haemodialysis patients by comparing its results with those of standard-of-care, blood-based laboratory methods used in different clinical sites.

## MATERIALS AND METHODS

### Patient selection

This was a prospective, open-label, non-randomized study comparing the results from the SmartPatch with the laboratory results during the same haemodialysis session. Adults with capacity to consent and who were receiving haemodialysis via an arteriovenous fistula (AVF) or arteriovenous graft (AVG) in either upper limb were enrolled in this study. Patients who were experiencing discomfort on the SmartPatch placement site, pregnant, participating in another study that would potentially affect either study's data quality, or, in the primary investigator's opinion, at an additional risk from participation were excluded from this study.

### Study procedure

All the study sites had approvals from their local ethics committee or institutional review board, and the study was conducted in accordance with the Declaration of Helsinki. All subjects that met eligibility criteria and agreed to participate in the study were provided with written informed consent prior to participation, along with a patient information sheet. The following information about each subject was obtained: general demographic information (date of birth, gender, height, weight, race, ethnicity, and skin pigmentation), health history, AVG/AVF access history, current access location, current medications/medication history, and pregnancy test result, if applicable. Participants’ skin tone was also assessed using the Massey scale [20]. The Massey scale provides a numerical label of skin pigmentation from 1 to 10 (light to dark).

All adverse events and device malfunctions were recorded. Safety-related events were categorized and documented in accordance with the Code of Federal Regulations, FDA research policy, and all current applicable regulations.

The SmartPatch was placed over the AV access site prior to the pre-dialysis blood draw. The SmartPatch was programmed to take readings at intervals which coincided with the pre- and post-dialysis session blood draws. After the pre-dialysis blood draw was performed, the subject underwent their routine dialysis session. A post-dialysis blood draw was performed once dialysis was completed and just before the SmartPatch was removed. Each blood sample was analysed for Hb and Hct values by the hospital's CLIA (Clinical Laboratory Improvement Amendments)-certified laboratory. Each subject could participate in up to four dialysis sessions for the study. This allowed up to eight separate pre- and post-dialysis data points from each subject to be included in the analysis.

### Study device

**Figure 1:**
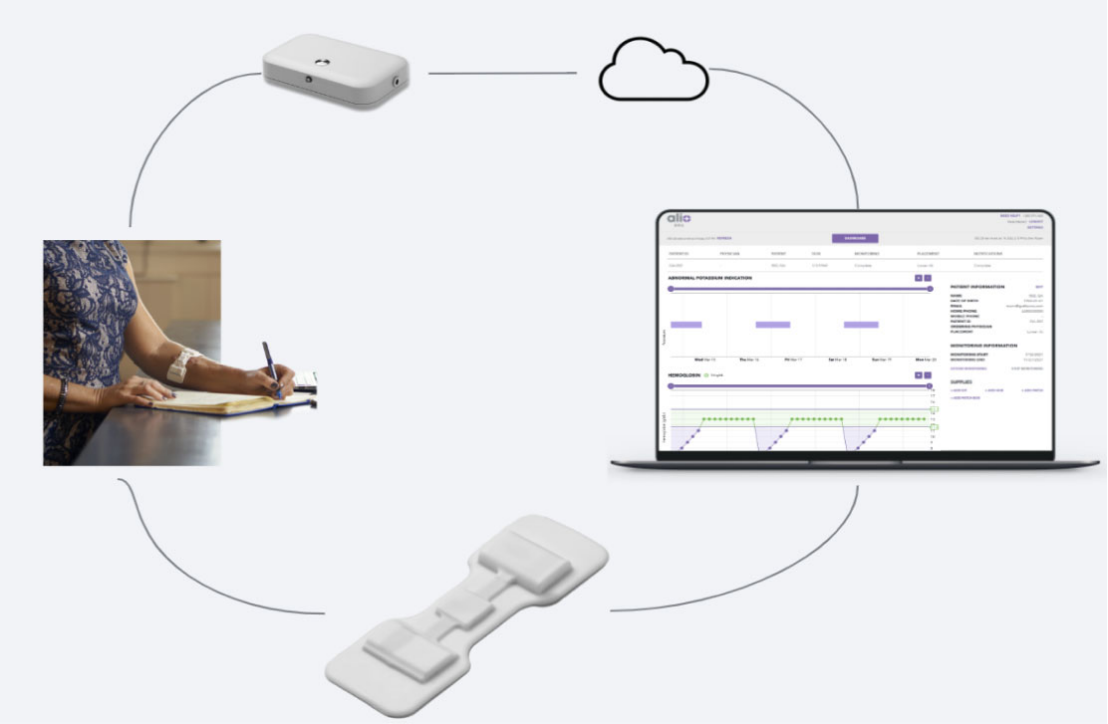
The SmartPatch, hub, and clinician portal.

The SmartPatch is a continuous, non-invasive, FDA-cleared remote monitoring device that is worn directly over an upper extremity AV fistula or AV graft. It utilizes Bluetooth to relay raw, encrypted HIPAA (Health Insurance Portability and Accountability Act)-compliant data via a cellular-enabled hub to a cloud-based server where it is processed and analysed using proprietary algorithms and subsequently displayed securely via a browser-based clinician portal. As shown in Fig. 1, the server decrypts and stores the SmartPatch data without associating any of the data with a subject's personal health information.

The platform is simple to use and requires minimal training for the clinical staff, and in this study all the SmartPatch devices were applied by trained staff. The SmartPatch is water-resistant (IP63) and is typically changed at 14-day intervals. The data are always accessible to the clinical team via the HIPAA-compliant web-based portal, and notifications can be automatically sent to a designated provider when a clinically relevant abnormality is detected.

### Data processing and signal quality

The Alio SmartPatch and cloud infrastructure have been previously described by Miller *et al*. [19]. Briefly, SmartPatch readings were processed using commonly accepted photoplethysmography data processing routines and additional proprietary signal processing and prediction algorithms. The Alio SmartPatch comprises multiple optical source detector combinations, which are leveraged to ensure robust readings. Multiple channels are synthesized into a single prediction following signal processing and quality checks. These checks include signal-to-noise assessment, data density assessment, patch-on-body detection, and motion rejection. Measurements were excluded if data recording failed in any of these checks.

The SmartPatch readings were obtained as close to the reference blood sample measurements as possible. All SmartPatch readings and reference blood draw measurements were taken within 5 min of each other to minimize any time-related shifts in Hb or Hct values. To avoid the confounding effects of filtration and fluid removal during haemodialysis, which can cause an interim period of significant instability of Hb and Hct levels, all readings taken during dialysis were excluded from analysis. The SmartPatch data analysis included two high-quality SmartPatch readings obtained closest in time to each blood draw (before and after the dialysis session); a maximum of four SmartPatch readings for each patient per dialysis session were obtained.

### Statistics

The primary endpoint of this study was a performance goal of the SmartPatch to measure Hb and Hct levels to within 95% confidence interval limits of agreement (LoA) of −6 to 6 percentage points (% Hct) for Hct, and −2 to 2 g/dL for Hb. Sample size calculation was performed prior to the commencement of the study, and a minimum sample size of 80 paired data points from a minimum of 20 subjects was determined to provide 80% power in measuring Hct and Hb levels within their respective LoA.

For Hb and Hct, a pooled root mean square difference (RMSD) across participants was calculated using the following equation:


\begin{eqnarray*}
\mathit{RMSD} = \sqrt {\frac{{\sum\nolimits_{i = 1}^N {{{{({{x}_i} - {{{\hat{x}}}_i})}}^2}}}}{N}}
\end{eqnarray*}

where *i* is variable *i, N* is the number of non-variable data points, *x_i_* is the number of actual observations (reference device) and *x*_1_ is predictions (SmartPatch).

Simple regression analysis and Bland–Altman analysis for repeated measurements were performed for Hb and Hct data. Bias (mean absolute difference of the non-dependent and dependent variables) and LoA were calculated using the Python stats models package, version 0.12.1.

## RESULTS

A total of 125 patients from two geographic regions—the USA (three sites) and the Kingdom of Jordan (one site)—were enrolled in this study. The subjects were enrolled between 23 May 2022 and 15 July 2022. Three patients withdrew from the study before completion: two prior to placement of the SmartPatch and one due to dizziness during the first visit.

Thus, the total study sample was 122 patients, of which 116 were dialysed using native AVF and six using AVG. Due to the small sample size of patients (4.91%) with AVG, they were excluded from the final analysis. Each patient was allowed to participate in up to four dialysis sessions, which yielded 282 SmartPatch readings that were used for analysis.

The mean age of the cohort was 56.1 ± 13.4 years (Table 1), and the baseline population characteristics are shown in Table 2.

**Table 1:** Baseline population characteristics stratified by sex [mean (SD)].

	Men*N* = 75	Women *N* = 41	Total *N* = 116	*P*-value
Age, years	55.2 (±12.9)	57.6 (±14.3)	56.1 (±13.4)	0.308

**Table 2:** Baseline population characteristics.

Attribute	*N* = 116 *n* (%)
Race	
Middle Eastern, Arab	63 (54.3)
Caucasian	28 (24.1)
Black or African American	18 (15.5)
American Indian or Alaska Native	5 (4.3)
Native American or Other Pacific Islander	2 (1.7)
Medical history	
Hypertension	98 (84.5)
Diabetes: all types	60 (51.7)
Subtypes: Type I, Type II, unknown	6 (5.2), 46 (39.7), 8 (6.9)
Anaemia	57 (49.1)
Heart disease	26 (22.4)
Heart failure	15 (12.9)
Valvular heart disease	8 (6.6)
Sleep apnoea	6 (4.9)
COPD	6 (4.9)
Atrial fibrillation	6 (4.9)
Implant: pacemaker or AICD	3 (2.5)
Coronary revascularization (stent, CABG)	2 (1.6)
Pulmonary hypertension	1 (0.8)
Polycystic kidney disease	1 (0.8)

**Table 3:** Concordance matrix for Hct.

		Reference device			
Female	*N* = 91	≤28%	>28% to <36%	Normal	>44%
SmartPatch	≤28%	0	2	0	0
	>28% to <36%	5	69	4	0
	Normal	0	3	6	1
	>44%	0	0	1	0
		Reference device			
Male	*N* = 191	≤30%	>30% to <41%	Normal	>50%
SmartPatch	≤30%	12	24	0	0
	>30% to <41%	25	126	1	0
	Normal	0	1	2	0
	>50%	0	0	0	0

**Table 4:** Concordance matrix for Hb.

		Reference device			
Female	*N* = 91	<9 g/dL	≥9 to <12.3 g/dL	Normal	>15.3 g/dL
SmartPatch	<9 g/dL	2	5	0	0
	≥9 to <12.3 g/dL	4	71	2	0
	Normal	0	2	4	1
	>15.3 g/dL	0	0	0	0
		Reference device			
Male	*N* = 191	<10 g/dL	≥10 to <14 g/dL	Normal	>17.5 g/dL
SmartPatch	<10 g/dL	39	49	0	0
	≥10 to <14 g/dL	21	10	2	0
	Normal	0	0	0	0
	>17.5 g/dL	0	0	0	0

**Figure 2:**
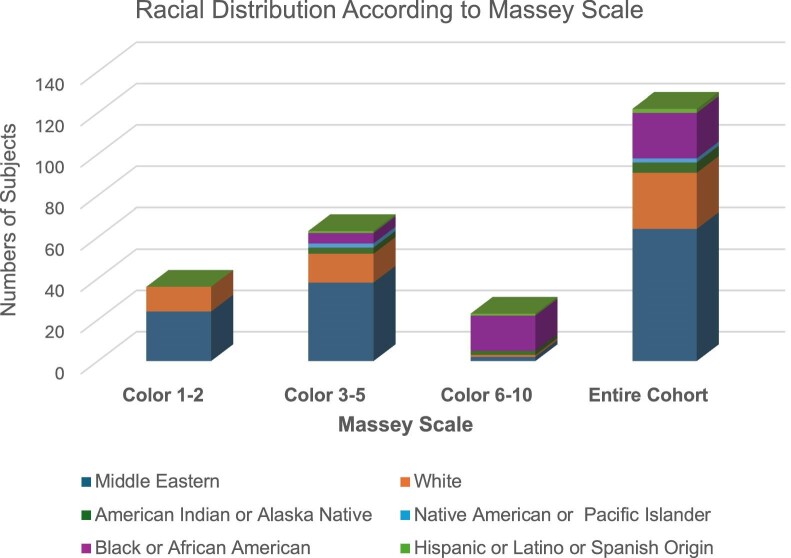
Distribution and racial make-up of skin tone.

Skin colour was classified using the Massey scale: light tones (1) to very dark tones (10). For this analysis, the pigmentation scale was divided into three categories: light (types 1 and 2), medium (types 3–5), and dark (types 5–10). A one-way analysis of variance was performed across each skin tone of the study cohort measured using the Massey scale (Fig. 2). No significant difference was found (*P* > 0.05).

All 116 patients used a native AVF for dialysis access. The left upper arm was the site most often used for access in 65 subjects (56.0%), followed by the left lower arm in 28 (24.1%), the right upper arm in 16 (13.8%), and the right lower arm in 7 (6.0%).

**Figure 3:**
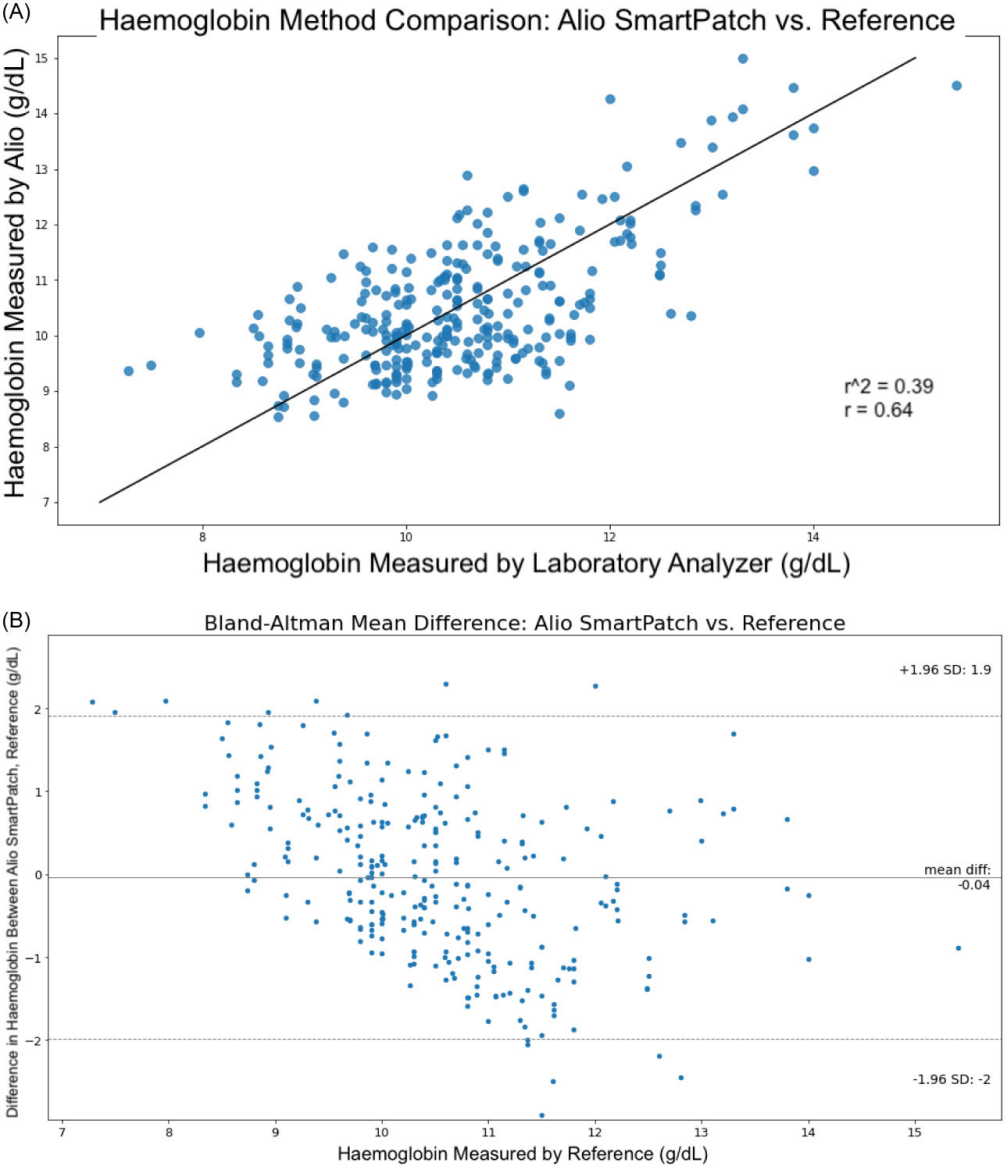
(A) Coefficient of determination (*r*^2^), correlation coefficient (*r*), and (B) Bland–Altman RMSD of non-invasive SmartPatch versus laboratory reference device for Hb (g/dL).

Haemoglobin measured by the reference device ranged from 7.28 to 15.4 g/dL, with a mean of 10.49 g/dL and median of 10.4 g/dL. A pooled RMSD of 1.0 g/dL was achieved across all participants. The 95% confidence interval LoA was −2.00 to 1.86 g/dL. The Pearson *r* correlation of 0.64 was statistically significant (*P* < 0.00001) (Fig. 3A). Figure 3B shows the Bland–Altman mean difference and Pearson *r* plots of all subjects. Bias as shown on the Bland–Altman plot corresponds to an Hb mean difference of −0.04 g/dL.

**Figure 4:**
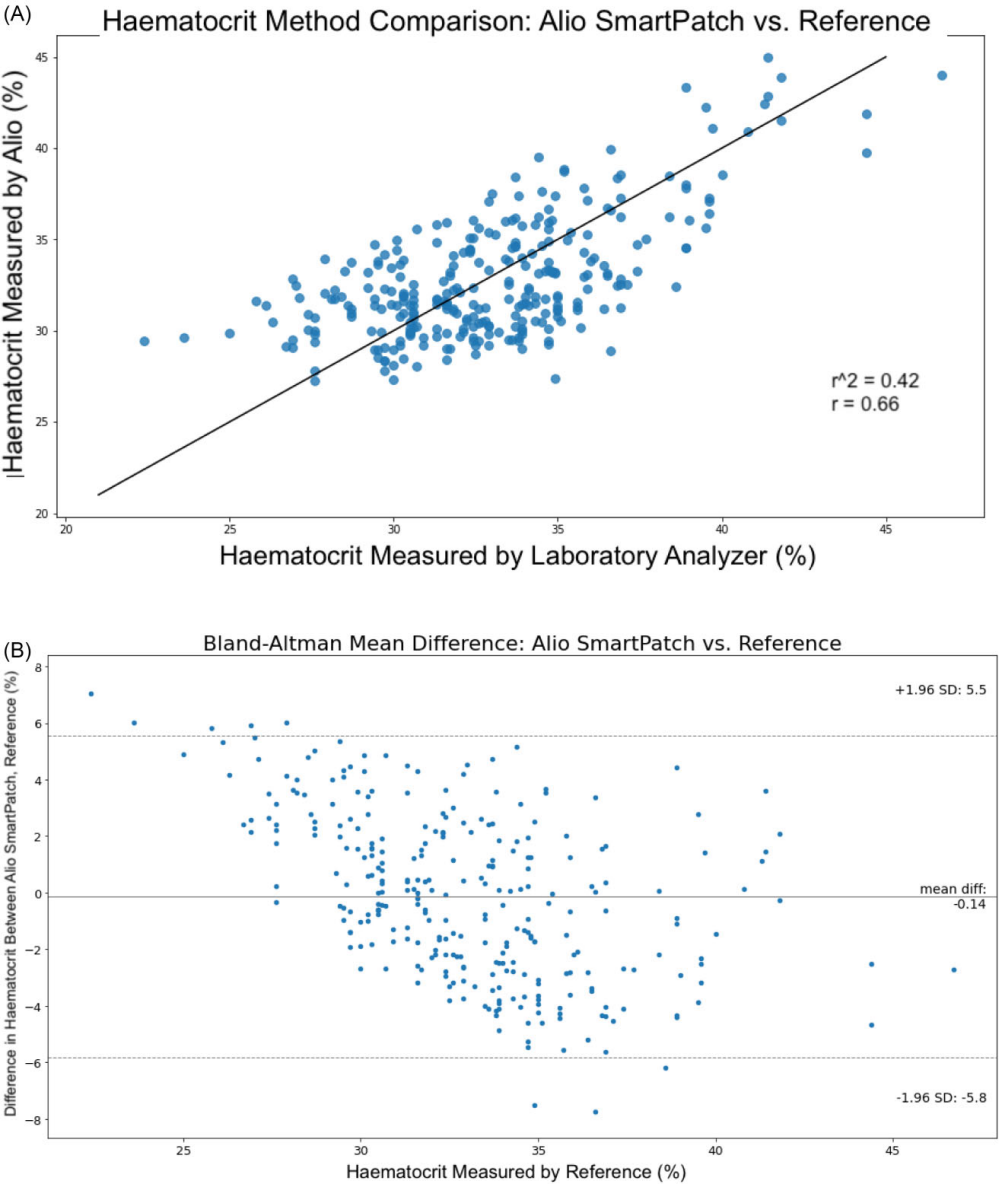
(A) Coefficient of determination (*r*^2^), correlation coefficient (*r*) and (B) Bland–Altman RMSD of non-invasive SmartPatch versus laboratory reference device for Hct (%).

Haematocrit measured by the reference device ranged from 22.4 to 46.7%, with a mean of 32.77% and median of 32.6%. A pooled RMSD of 2.91 percentage points of Hct (% Hct) was achieved across all participants. The 95% confidence interval LoA was −5.84 to 5.53% Hct. The Pearson *r* correlation of 0.66 was statically significant (*P* < 0.00001). Bias as shown on the Bland–Altman plot corresponds to an Hct mean difference of −0.14% (Fig. 4A and B).

SmartPatch Hb and Hct measurements achieved the primary endpoint of the study, which was a performance goal to measure Hb and Hct to within 95% confidence interval LoA of −6 to 6 percentage points of Hct and −2 to 2 g/dL for Hb at 80% power.

The normal Hct for adult females and males is generally 36–44% and 41–50%, respectively [21]. Figure 5 shows the values reported by the SmartPatch compared with the contemporaneous values reported by the reference sample. The SmartPatch is indicated by the FDA as an adjunct device and therefore necessitates a confirmatory blood draw before any clinical actions are taken. The red areas represent areas of severe disagreement, which could possibly lead to unnecessary blood draws. No areas of severe disagreement were noted in this evaluation. The dark green areas represent concordance between the two measures, and the light green areas represent mild disagreement that may be caused by patient values at the edges of the ranges shown in the figures. When focusing on the low end (anaemia), mild disagreements may or may not elicit a blood draw.

Similarly, normal Hb for adult females and males is generally 12.3–15.3 and 14–17.5 g/dL, respectively. Using the same scheme as that in Fig. 5, Fig. 6 shows the data displayed for Hb [22]. There is excellent concordance between the SmartPatch and reference sample with no areas of severe disagreement.

Our analysis shows concordance in the majority of the measures (411/564, 73%), with mild disagreement in a small cohort (153/564, 27%), and no severe disagreement between the SmartPatch and the reference readings was noted.

## DISCUSSION

In this study, we compared the SmartPatch Hb and Hct measurements with laboratory analysis of whole blood samples. The LoA for Hb measurements were −2.00 to 1.86 g/dL, and for Hct measurements were −5.84 to 5.53%. These findings underscore the precision of the SmartPatch in monitoring both Hb and Hct levels, demonstrating tighter LoA than for other non-invasive devices.

To put these results into perspective, a similar analysis conducted by Hiscock *et al*. assessed various Hb measuring devices against traditional laboratory results. Their study found that non-invasive Masimo devices (Rad-7 and Pronto-7) displayed a wider LoA of ∼±3 g/dL, whilst the minimally invasive HemoCue devices showed an LoA ranging from −1.25 to 1.41 g/dL. These comparative findings, detailed alongside our results in Table 5, appended from the article of Hiscock *et al*., highlight the enhanced accuracy and reliability of the SmartPatch for clinical use in Hb monitoring [23].

**Table 5:** Comparison of LoA for Hb measurements [23].

Method	Type	SD of meandiff (g/dL)	95% LoA (g/dl)
Hemocue	B-Haemoglobin	0.73	−1.50 to 2.56
	201+	0.80	−1.96 to 2.16
	Unknown	0.52	−1.29 to 1.03
	Overall	0.64	−1.25 to 1.41
Pulse CO-Oximetry	Pronto-7	1.2	−2.90 to 3.26
	Radical-7	1.5	−3.26 to 3.04
	Overall	1.42	−2.97 to 3.04
Alio Platform	SmartPatch	1.0	−2.00 to 1.86

The transformation seen in diabetes management through continuous glucose monitoring (CGM) systems underscores the impact of innovative technologies on chronic disease management. CGMs facilitate continuous, minimally invasive, and remote monitoring of glycaemic levels, thus significantly enhancing diabetes care by improving patient autonomy and optimizing therapeutic interventions [24]. Similarly, the SmartPatch's ability to accurately measure Hb levels compared with reference blood samples holds the potential to revolutionize anaemia management in CKD, especially for patients with ESKD.

The SmartPatch's capability to monitor Hb and Hct levels frequently, remotely, and in a non-invasive manner represents an advancement over traditional laboratory tests and other available devices, offering a new approach to managing the complexities of anaemia in CKD patients. As previously discussed, the necessity for administering supplementary ESAs is critical to compensate for the insufficient endogenous erythropoietin in CKD patients, which complicates the stabilization of Hb levels [6, 9–11]. Highlighting this complexity, the latest KDIGO guidelines on anaemia management emphasize that ‘the accuracy of projection (extrapolation) increases with the number of contributing data points, and the frequency of Hb monitoring is likely to be an important determinant of the accuracy of ESA dose adjustment’ [5]. However, despite extensive reviews and evaluations, the optimal interval for Hb testing remains undefined [25].

These higher-fidelity trend lines from the SmartPatch may be used to improve the precision of ESA dosing intervals. For example, most ESAs require >2 weeks to begin to influence Hb levels visibly [26]. Patient responses to ESAs can vary significantly due to individual factors, such as age, residual renal function, and dietary iron consumption [9, 12]. This variability makes managing dosing regimens particularly challenging. The high sampling resolution of the SmartPatch enables tracking of an individual's Hb levels and should enable a higher degree of optimization of the dosing intervals at the individual patient level. Therefore, higher-resolution tracking of Hb values could enable more precise dosing of ESA therapy and thus could potentially result in more efficient use of this costly therapy, while simultaneously personalizing the dosing strategy for every patient to minimize the time required to stabilize Hb levels [27–30]. Further clinical studies are needed to investigate this potential application of the device.

The increasing need for improved anaemia management coincides with the trend towards expanding home dialysis. As the prevalence of dialysis rises and home dialysis therapies improve, there has been a growing shift away from traditional in-centre dialysis settings. In 2019, the US Department of Health and Human Services launched an initiative called ‘Advancing American Kidney Health’ with the goal of increasing the number of home dialysis recipients to 80% by 2025 [31]. According to the most recent report by the United States Renal Data System in 2022, current home dialysis usage stands at 13.3% [32], indicating substantial room for growth to meet these objectives. The SmartPatch supports this transition by providing a ‘set it and forget it’ solution for real-time, remote monitoring that does not require active patient engagement beyond initial setup. As the only FDA-cleared device that offers such capabilities, the SmartPatch is uniquely positioned to facilitate a shift towards more patient-centred, home-based dialysis care.

## CONCLUSION

The FDA-cleared SmartPatch stands to transform anaemia management in CKD much like CGM systems have revolutionized diabetes care. By providing longitudinal, non-invasive, and accurate monitoring of key blood parameters, the SmartPatch enables a nuanced and responsive approach to managing anaemia. This capability not only promises to improve individual patient outcomes but also supports a shift towards a more patient-centred healthcare model. The SmartPatch offers a promising future where ESA therapy is as dynamically managed as insulin therapy today. As CKD prevalence increases, technologies like the SmartPatch will be important in effectively managing complex chronic conditions, ensuring that patients receive the best possible care.

There are limitations to this evaluation. This analysis was conducted assessing the SmartPatch's accuracy against the gold standard invasive blood draws, over a finite collection period during a patient's dialysis session. Shifts in Hb and Hct that occur in between dialysis sessions were not evaluated in this study. Future longitudinal studies are planned to evaluate the SmartPatch over several months, including inter-dialysis periods, to assess its potential in transforming anaemia patient management.

Subjects with known haemoglobinopathies, such as thalassemia or sickle cell disease, were excluded to avoid confounding effects, given that these conditions can affect the optical properties of blood and impair the SmartPatch sensor's performance. Additionally, the data analysed were for a SmartPatch placed over the AVF. Subjects with an AVG were not analysed due to the lower number of AVGs enrolled. Future studies will assess the SmartPatch's accuracy on AVGs as well as other anatomical locations. Lastly, although efforts were made to include a diverse demographic population with different skin types and skin tones, the performance of the device on all skin types over time will need further study. This validation study provides a promising outlook on the accuracy of the SmartPatch in remote monitoring technologies for non-invasive, frequent monitoring of anaemia to radically impact the typical paradigm of care for patients with chronic diseases. Addressing the identified limitations through further longitudinal randomized clinical trials will strengthen the evidence base and support broader clinical adoption, thereby continuing to assess the potential impact of the Alio platform on clinical decision-making and outcomes.

## Data Availability

The data underlying this article will be shared on reasonable request to the corresponding author.
